# Systematic Review Shows Tele-Rehabilitation Might Achieve Comparable Results to Office-Based Rehabilitation for Decreasing Pain in Patients with Knee Osteoarthritis

**DOI:** 10.3390/medicina57080764

**Published:** 2021-07-28

**Authors:** Tamila Latif-Zade, Brian Tucci, Danna Verbovetskaya, Elizabeth Bialkin, Brian Ng, Stephan Heddon, Jean-Philippe Berteau

**Affiliations:** 1 Department of Physical Therapy, College of Staten Island, City University of New York, Staten Island, NY 10314, USA; tamila.latifzade@cxi.csi.cuny.edu (T.L.-Z.); brian.tucci@cxi.cuny.csi.edu (B.T.); Dana.Verbovetskaya@cxi.csi.cuny.edu (D.V.); Elizabeth.Bialkin@cxi.csi.cuny.edu (E.B.); Brian.Ng@cxi.csi.cuny.edu (B.N.); stephanheddon@yahoo.com (S.H.); 2 New York Centre for Biomedical Engineering, City College of New York, City University of New York, New York, NY 10031, USA; 3 Nanosciences Initiative, Advanced Science Research Center, City University of New York, New York, NY 10031, USA

**Keywords:** knee osteoarthritis, tele-rehabilitation, exercise

## Abstract

*Background and Objectives* This systematic review aims to evaluate the efficacy of Tele-Rehabilitation for decreasing pain in patients with knee osteoarthritis (OA). *Materials and Methods*: Following the guidelines of Preferred Reporting Items for Systematic Reviews and Meta-Analyses (PRISMA), three electronic databases (CINAHL, PubMed, PEDro), along with the addition of grey literature, were used to collect information. Randomized control trials (RCTs) comparing tele-rehabilitation (TR) to office-based-rehabilitation (OB) were critically appraised using the 2005 University of Oxford Standard. A total of 139 articles (PubMed = 132, CINAHL = 5, PEDro = 0, grey literature = 2) were acquired. *Results*: After the screening, three RCTs were included in our review. Their results show no statistically significant differences between TR and OB intervention. Furthermore, their results showed an overall reduction in pain in both groups from the baseline to the end of the study. However, each intervention’s clinical efficiency was dependent on the exercise protocol itself and not on the method of delivery. There is a potential ceiling effect to the amount of therapy a patient can receive in which additional therapy would no longer lead to improved recovery. *Conclusions*: Our review suggests evidence that TR’s efficacy is similar to that of OB for improvement of WOMAC (Western Ontario and McMaster Universities Osteoarthritis Index) score parameters in patients suffering from knee OA.

## 1. Introduction

Osteoarthritis (OA) is a musculoskeletal disease that induces degeneration of cartilage, subchondral bone, and joint and tissue mechanics alterations [1,2,3,4,5]. Affecting more than 32.5 million adults in the United States alone, OA is the second most costly health condition treated in US hospitals [6], accounting for $16.5 billion, or 4.3%, of the combined cost of all hospitalizations [7]. Regarding knee OA, it is considered one of the most prevalent causes of disability and pain in older individuals [1,8,9]. For instance, among adults 60 years and older, its prevalence is approximately 10% in men and 13% in women. It is expected to rise with the increasing elderly population and obesity rate [2].

Several rehabilitation methods have been proven effective at decreasing pain and OA progression, such as neuro-muscular electrical stimulation [10] and exercise programs [11,12,13,14]. For instance, strengthening the muscles around the knee relieves some of the stress on the joint itself, allowing for the slowing down of the degeneration of the articular cartilage and the vicious circle of degradation. These interventions are generally delivered in an office-based clinical setting where rehabilitation practitioners guide the patients and monitor the intervention’s effectiveness. During the global COVID-19 pandemic, tele-rehabilitation (TR)—the use of technologies to provide rehabilitation services to people in their homes—has been in increasing demand [15,16] because of safety reasons. When physical distancing is mandatory, a guided exercise program is one of the only alternatives for performing or continuing the rehabilitation program. Unfortunately, there is currently a lack of knowledge regarding (i) the efficiency of TR for knee OA and (ii) literature standards to help rehabilitation practitioners.

This systematic review aims to determine the effectiveness of TR in the treatment of knee OA with a focus on exercise. We hypothesized that an evidence-based progressive, individualized home exercise program for people with knee OA would bring the same efficiency whether it is delivered in clinics or remotely. To test our hypothesis, we reviewed studies where the intervention was primarily exercise, and the patients’ pain levels were measured as an outcome.

## 2. Materials and Methods

For this review, we used PRISMA guidelines and set up our PICO (Population, Intervention, Comparison, Outcome) as follows: the population was patients at least 35 years of age who have knee OA; the intervention was tele-rehabilitation; the comparison was office-based rehabilitation; the outcome was pain level evaluated with the WOMAC score (Western Ontario and McMaster Universities Osteoarthritis Index).

Data Sources and Searches: We searched—between 15–30 June 2020—through three databases, including CINAHL, PubMed, and PEDro, and in grey literature. Our MeSH words entrance were: [knee osteoarthritis] OR [osteoarthritis of the knee] OR [osteoarthritis of knee]—AND 15 June and 30 June [Tele-Rehabilitation] OR [tele-rehabilitation] OR [virtual rehabilitation] OR [remote rehabilitation]—AND [pain]—AND [exercise].

Study Selection: Using those MeSH words, we obtained 132 articles from PubMed, five articles from CINAHL, 0 from PEDro, and we brought two additional articles upon searching through grey literature. After duplicates were removed, we ended up with 138 articles. We further screened these articles with our inclusion and exclusion criteria (listed in Table 1) and ended up with three eligible articles for critical appraisal. Both of our investigators (TL and EB) conducted critical appraisals for each of the articles.

Data Extraction and Quality Assessment: To screen for the risk of bias, two researchers (EB and TL) performed critical appraisals on all the retrieved abstracts. They screened for internal validity, equal treatment of patients aside from the allocated intervention, and unbiased practices based on the 2005 Oxford standard [17]. If there were any disagreements in the texts used, there would be a discussion and consultation with a senior researcher (JPh B.).

Data Synthesis and Analysis: A summary of results from the eligible studies is compiled in Table 2 by listing the authors, interventions for experimental and control groups, group sample sizes, outcome measures, and the relative risk reduction. Relative risk reduction (RRR) was calculated following the 2005 University of Oxford guidelines [17], where a RRR > 1 indicates that the treatment increased the risk of the outcome according to the following formula:

Relative Risk Reduction = (Outcome in Treatment group)/(Outcome in Control group)

## 3. Results

**Figure 1:**
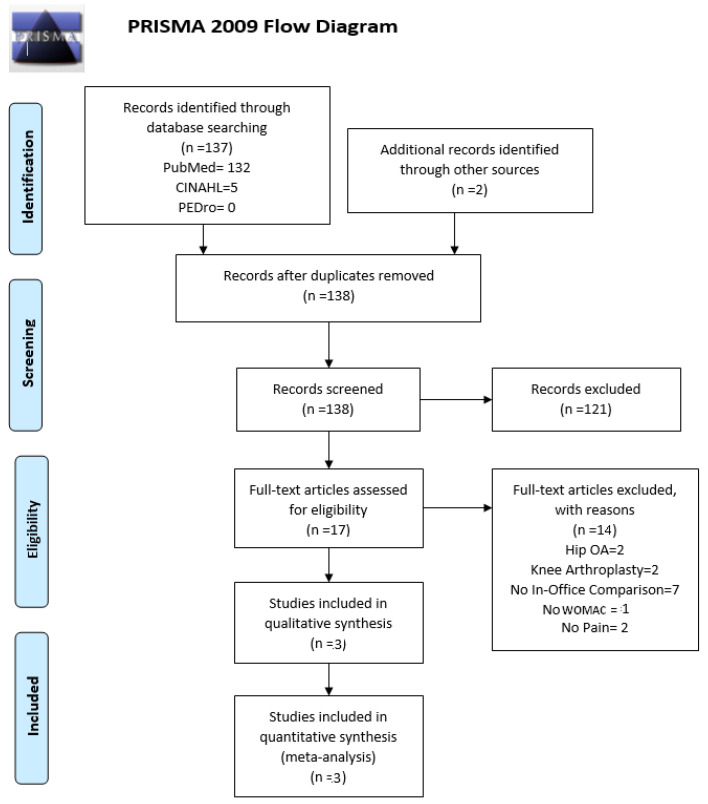
Graphical representation of our PRISMA flowchart. OA: osteoarthritis; WOMAC: Western Ontario and McMaster Universities Osteoarthritis Index.

Following the guidelines of PRISMA, a total of 139 articles (PubMed = 132, CINAHL = 5, PEDro = 0, Grey literature = 2) were acquired. One duplicate was removed, leaving 138 articles. After a preliminary screening of the articles, 121 were excluded as they did not pertain to our topic (the paper did not address the question at all, either in their title or in the abstract). We then screened the remaining 17 studies using our inclusion and exclusion criteria and were left with three articles, critically appraised and used in our systematic review. The three studies were Randomized Control Trials (level 1b). There were no studies excluded due to the age or gender of participants. A total of 526 participants were analyzed from those three articles. Figure 1 is a graphical representation of our PRISMA flowchart. Table 2 represents a summary of the findings from the three papers analyzed. Table 3 represents a summary of the risk of bias within the three studies. Our review of the three studies showed that they found (i) statistically significant decreases in pain levels from the baseline to the end of the study and (ii) no statistically significant differences between TR and OB in all three studies.

**Figure 2:**
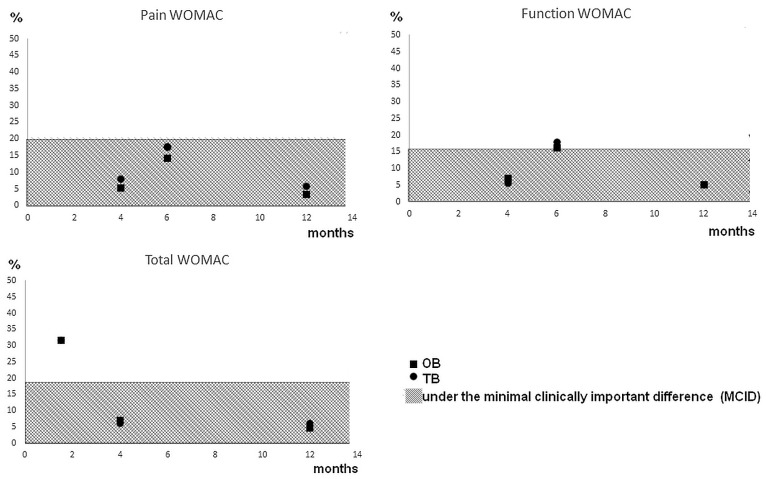
Percentage of changes for each WOMAC score evaluated and the associated MCID.

Regarding Bennell et al. [20], they compared six months (five sessions) of OB and (up to six sessions of) telephone delivered TR. The RRR shows a decrease of between 14% and 17% for both scores and groups at six months (Table 2). The researchers did a follow up at (i) 12 and (ii) 18 months, showing a mean within-group difference from baseline of (i) TR: 3.8, OB: 3.1 for pain WOMAC scale and TR: 14, OB: 12.9 for function WOMAC scale, and (ii) TR: 3.7, OB: 4.2 for pain WOMAC scale and TR: 15.1, OB: 13.9 for function WOMAC scale. When testing for statistical difference between TR and OB, both the pain and functional WOMAC scores at months 6, 12, and 18 were not statistically different. Regarding Allen et al. [18], they compared OB with internet-delivered TR (up to eight sessions) and patients on a waitlist (control, CL). The RRR shows a decreased spread between 3 and 9% for the three scores and groups at 4 and 12 months (Table 2). Total, functional, and pain WOMAC were not statistically different after four months or 12 months. Regarding Azma et al. [19], they compared OB with TR after six weeks (18 sessions). The RRR shows a decrease of between 31.8% and 31.5% for both groups (Table 2). The researchers also did a follow-up at (i) one and (ii) six months, showing a mean within-group difference of total WOMAC scale from a baseline of (i) TR: 32.30, OB: 32.38, and (ii) TR: 32.55, OB: 32.02. No significant difference was found between OB and TR groups. In addition to this, we plotted (Figure 2) the percentage of changes for each WOMAC score evaluated and the minimal clinically important difference (MCID) associated with each one.

## 4. Discussion

After screening, three RCTs were included in our review, and our critical appraisal detected a low risk of bias for each study. Although we showed statistically significant decreases in pain levels from the baseline to the end of the three studies, suggesting that the interventions were successful. One of the authors compared their results to the MCID. The MCID is the smallest change in score that a patient will perceive in their clinical state, which would indicate a change in the patient’s management. Recent evidence for knee OA has depicted that the MCID is 17% for total WOMAC score (i.e., the change is not due to uncertainty in the score after this threshold), 21% for WOMAC pain, and 16% for WOMAC function [21]. In relation to the MCID, we plotted (Figure 2) the percentage change for each WOMAC score evaluated and the associated MCID.

Our figure shows that the Azma et al. study [19] showed the best TR and OB intervention benefits. They passed the threshold of minimum important change (roughly 30% for both interventions when 17% is needed for total WOMAC score). When looking at Bennell et al. [20], their interventions reached the MCID for the WOMAC Function score (roughly 17% for both interventions when 16% is needed) but not for the WOMAC pain score (between 14% and 17% when 21% is required). Regarding the study by Allen et al. [18], none of their interventions reached any of the MCID recommended (between 5% and 8% when 16.5% is the minimum needed). One of the main challenges for comparing the studies is that the exercises prescribed were not deeply detailed and non-uniform. It is the same for delivery methods, including video clips, pamphlets, and coaching through the telephone. Another study done by Odole and Ojo [22] was found to support TR’s practical use. Although they showed a reduction in pain with both TR and OB, this study used the visual analogue scale (VAS) to measure pain intensity in patients instead of the WOMAC, making it difficult to compare our findings. However, the MCID for VAS is 22.6 units, and both TR and OB showed a decrease above 30 units after six weeks of intervention and without statistical difference between the groups.

Regarding comparing the TR and OB groups for each of the three studies, two studies (Bennell et al. [20] and Allen et al. [18]) had the same protocol regarding therapeutic exercise for both groups, and the difference was the method of delivery—with no differences in medication between groups. For instance, Bennell et al. [20] used an evidence-based, progressive, individualized exercise program (comprising 4–6 lower-extremity exercises performed three times per week). Regarding Allen et al. [18], the exercises primarily targeted the quadriceps, hamstrings, and gluteal muscles, while the aerobic recommendations comprised a progressive walking program. All exercises were to be performed five times per week (consistent with the current standard of care and clinical practice guidelines). On the contrary, the Azma et al. study [19] had some additional interventions in the OB program, such as various passive physiotherapeutic modalities (e.g., electrical stimulation, ultrasound) but no other medication. 

Regarding clinical efficiency, we show that the best intervention for decreasing the WOMAC score was performed in the study of Azma et al. [19]. They used a program where the participants had strengthening, endurance, flexibility, and active range of motion exercises. In addition to these exercises, they were given a heat pack on the knee for 20 min before exercise. The other protocol reaching MCID was Bennell et al. [20] The authors listed the number of exercises for the lower extremity and how they catered to the patients based on their assessment. Furthermore, their results show that participants, even after receiving telephone-delivered coaching sessions every month in addition to OB treatment, did not gain minimum significant change for pain at 12 and 18 months when compared to participants who just received OB treatment. Thus, adding TR at these periods to treat pain and functional issues did not decrease those symptoms, respectively. We hypothesize that this may be due to a ceiling effect of the amount of therapy a patient can receive beyond which additional treatment would no longer lead to improved recovery. There are some limitations to our systematic review. We acknowledge that (i) we focused on the pain parameter with a single index test and (ii) it will be of interest to perform analyses like sensitivity or subgroup analyses—however, most studies lack supplementary datasets that help in performing metanalyses. In addition, while the inclusion of participants was mainly based on the American College of Rheumatology criteria, the level of OA was never mentioned. While the three studies used an evidence-based, progressive, individualized home exercise program with different variations in length and follow-up, one study by Azma et al. [19] used additional treatment modalities for the OB intervention.

Although we found only three RCTs after the systematic review process, we believe that our result is still a piece of evidence showing that tele-rehabilitation might achieve comparable results to office-based rehabilitation for decreasing pain in patients with knee osteoarthritis. Indeed, our review shows that none of the studies we included showed statistical differences between TR and OB intervention regarding pain measured parameters. Furthermore, we evaluated the clinical efficiency of each intervention, and our study shows that the method of delivery did not impact it. To summarize, whatever the intervention used, none of the OB interventions provide better results than the TR. Thus, we recommend TR’s use for providing easier access to treatment for patients with knee OA when access to clinical practice is not possible.

## 5. Conclusions

Our review suggests evidence for the same efficacy of TR compared to OB for improvement of WOMAC score parameters in patients suffering from knee OA. While our study supports exercises delivered through TR as an effective intervention for patients, we also show that the main factor for reaching a minimal clinically important difference threshold is the rehabilitation intervention’s content and not the delivery method. There is a potential ceiling effect of the amount of therapy a patient can receive in which additional treatment would no longer lead to improved recovery.

## Figures and Tables

**Table 1:** Inclusion and exclusion criteria.

Inclusion	Exclusion
Male or female	Knee arthroplasty
Randomized study	Osteoarthritis in other joints
Remote treatment	No-exercise treatments
Exercise	(e.g., injection therapy)
Age 35+	
Western Ontario and McMaster Universities Osteoarthritis Index	

**Table 2:** Summary of results from the eligible studies (tele-rehabilitation (TR); office-based rehabilitation (OB); control (CL); WOMAC (Western Ontario and McMaster Universities Osteoarthritis Index); relative risk reduction (RRR)).

Study	Interventions	n(Group) = Sample Size	Mean within-Group Difference from Baseline	Relative Risk Reduction	TR vs. OB (*p* Values)
Allen et al. [18]			Pain WOMAC-Month 4	Pain WOMAC-Month 4	0.20
	TR	n(TR) = 112	TR: 1.59	RRR TR: 7.9%	
	OB	n(OB) = 129	OB: 1.11	RRR OB: 5.5%	
	CL	n(CL) = 63	CL: 0.66	RRR CL: 3.3%	
			Pain WOMAC-Month 12	Pain WOMAC-Month 12	0.22
			TR: 1.15	RRR TR: 5.7%	
			OB: 0.7	RRR OB: 3.5%	
			CL: 0.64	RRR CL: 3.2%	
			Function WOMAC-Month 4	Function WOMAC-Month 4	0.33
			TR: 3.74	RRR TR: 5.5%	
			OB: 4.77	RRR OB: 7.0%	
			CL: 2.3	RRR CL: 3.4%	
			Function WOMAC-Month 12	Function WOMAC-Month12	0.93
			TR: 3.4	RRR TR: 5.0%	
			OB: 3.3	RRR OB: 4.8%	
			CL: 1.51	RRR CL: 2.2%	
			Total WOMAC-Month 4	Total WOMAC-Month 4	0.65
			TR: 6.06	RRR TR: 6.3%	
			OB: 6.73	RRR OB: 7.0%	
			CL: 3.37	RRR CL: 3.5%	
			Total WOMAC-Month 12	Total WOMAC-Month 12	0.51
			TR: 5.46	RRR TR: 5.6%	
			OB: 4.42 CL: 2.83	RRR OB: 4.6% RRR CL: 2.9%	
Azma et al. [19]			Total WOMAC-Week 6	Total WOMAC-Week 6	0.66
	TR	n(TR) = 27	TR: 31.79	RRR TR: 31.8%	
	OB	n(OB) = 27	OB: 31.54	RRR OB: 31.5%	
Bennell et al. [20]			Pain WOMAC-Month 6	Pain WOMAC-Month 6	>0.05
	Rehabilitation + TR	n(TR) = 84	TR: 3.5	RRR TR: 17.5%	
	OB	n(OB) = 84	OB: 2.9	RRR OB: 14.5%	
			Function WOMAC-Month 6	Function WOMAC-Month 6	>0.05
			TR: 11.8	RRR TR: 17.3%	
			OB: 11.4	RRR OB: 16.8%	

**Table 3:** Summary of the risk of bias within the three studies (screened for unbiased practices based on the 2005 Oxford standard [17].

Study	Was the Assignment of Patients to Treatments Randomized?	Were the Groups Similar at the Start of the Trial?	Aside from the Allocated Treatment, Were Groups Treated Equally?	Were All Patients Who Entered the Trial Accounted for?	Were Measures Objective or Were the Patients and Clinicians Kept “Blind” to Which Treatment Was Being Received?
Allen et al. [18]	Yes	**Unclear**	Yes	Yes	Unclear
Azma et al. [19]	Yes	Yes	Yes	Yes	Unclear
Bennell et al. [20]	Yes	Yes	Yes	Yes	Yes
